# Structural validation of the Self-Compassion Scale with a German general population sample

**DOI:** 10.1371/journal.pone.0190771

**Published:** 2018-02-06

**Authors:** Adina Coroiu, Linda Kwakkenbos, Chelsea Moran, Brett Thombs, Cornelia Albani, Sophia Bourkas, Markus Zenger, Elmar Brahler, Annett Körner

**Affiliations:** 1 Department of Educational and Counselling Psychology, McGill University, Montreal, Canada; 2 Department of Psychiatry, McGill University, Montreal, Canada; 3 Lady Davis Institute for Medical Research, Jewish General Hospital, Montreal, Canada; 4 Behavioural Science Institute, Clinical Psychology, Radboud University, Nijmegen, the Netherlands; 5 Department of Medical Psychology and Medical Sociology, Medical Center of the University of Leipzig, Leipzig, Germany; 6 Faculty of Applied Human Studies, University of Applied Sciences Magdeburg-Stendal, Stendal, Germany; 7 Integrated Research and Treatment Center (IFB) Adiposity Diseases - Behavioral Medicine, Medical Psychology and Medical Sociology, University of Leipzig Medical Center, Leipzig, Germany; 8 Department of Psychosomatic Medicine and Psychotherapy, Universal Medical Center Mainz, Mainz, Germany; Universitat Wien, AUSTRIA

## Abstract

**Background:**

Published validation studies have reported different factor structures for the Self-Compassion Scale (SCS). The objective of this study was to assess the factor structure of the SCS in a large general population sample representative of the German population.

**Methods:**

A German population sample completed the SCS and other self-report measures. Confirmatory factor analysis (CFA) in MPlus was used to test six models previously found in factor analytic studies (unifactorial model, two-factor model, three-factor model, six-factor model, a hierarchical (second order) model with six first-order factors and two second-order factors, and a model with arbitrarily assigned items to six factors). In addition, three bifactor models were also tested: bifactor model #1 with two group factors (SCS positive items, called SCS positive) and SCS negative items, called SCS negative) and one general factor (overall SCS); bifactor model #2, which is a two-tier model with six group factors, three (SCS positive subscales) corresponding to one general dimension (SCS positive) and three (SCS negative subscales) corresponding to the second general dimension (SCS negative); bifactor model #3 with six group factors (six SCS subscales) and one general factor (overall SCS).

**Results:**

The two-factor model, the six-factor model, and the hierarchical model showed less than ideal, but acceptable fit. The model fit indices for these models were comparable, with no apparent advantage of the six-factor model over the two-factor model. The one-factor model, the three-factor model, and bifactor model #3 showed poor fit. The other two bifactor models showed strong support for two factors: SCS positive and SCS negative.

**Conclusion:**

The main results of this study are that, among the German general population, six SCS factors and two SCS factors fit the data reasonably well. While six factors can be modelled, the three negative factors and the three positive factors, respectively, did not reflect reliable or meaningful variance beyond the two summative positive and negative item factors. As such, we recommend the use of two subscale scores to capture a positive factor and a negative factor when administering the German SCS to general population samples and we strongly advise against the use of a total score across all SCS items.

## Background

Compassion is a concept rooted in Buddhist philosophy, which represents a positive attitude whereby one can deeply care for one’s suffering (i.e., self-compassion) or be deeply moved by the suffering of another (i.e., compassion for others). In recent years, self-compassion has become a popular topic in psychology with numerous studies assessing the associations between self-compassion and other psychological variables. Meta-analyses on correlates of self-compassion, for instance, found positive associations with cognitive, psychological and affective well-being [1] and negative associations between self-compassion and physiological or psychological distress (i.e., anxiety and depression) [2]. The argument driving these studies is that self-compassion is a construct highly amenable to change, which can be used in psychotherapy to improve mental health outcomes. Interventions with a self-compassion component (e.g., compassionate mind training; mindful self-compassion program) have indeed been shown to increase life satisfaction in the general population [3] and to decrease depression, anxiety, and stress in community and clinical samples [3–7]. Gilbert [8] suggested self-criticism as the mechanism through which self-compassion acts to lower the emotional response to perceived threats. Self-criticism is a risk factor for various psychological difficulties, including mood disorders, anxiety, self-harm, suicide, and alcoholism [9]. In addition, studies have shown that self-compassion based interventions can be effective at improving self-compassion in various populations [3, 5, 10, 11]. Most studies to date assessed the construct of self-compassion using the Self-Compassion Scale (SCS) [12], with the majority of cross-sectional [2] and intervention studies [3, 10, 11] using a total summed SCS score as the measure of effect. Some studies have reported analyses involving SCS subscale scores [13, 14] or used both summed and subscale scores [5]. However, current methodological issues related to the measurement of self-compassion via the SCS make it difficult to contextualize the findings about the psychotherapeutic potential of self-compassion.

The Self-Compassion Scale was developed by Neff [12]. As per Neff’s definition, self-compassion encompasses three bipolar dimensions demarcated into six factors: a) the presence of self-kindness in contrast to self-judgment; b) a sense of common humanity as opposed to a sense of isolation; and c) mindfulness as opposed to over-identification [12, 15]. *Self-kindness* refers to being caring, understanding and accepting of oneself, becoming aware of, and being moved by one’s own suffering whereas *self-judgment* involves being harsh and extremely self-critical. *Common humanity* refers to the recognition that failure and hardship are shared human experiences, which foster connectedness to others rather than leaving one feeling *isolated* when faced with suffering. *Mindfulness* represents the acceptance of the present moment experience and involves taking an objective stance on one’s experience in order to gain perspective and to avoid *over-identification* with negative thoughts and emotions [15]. Neff [16] updated the definition of self-compassion to an interplay of positive and negative constructs, working as a dynamic system and includes “different ways that individuals emotionally respond to pain and failure (with kindness or judgment), cognitively understand their predicament (as part of the human experience or as isolating), and pay attention to suffering (in a mindful or over-identified manner).

The factorial structure of the original English SCS and its translations has not been consistently replicated and studies to date have yielded conflicting results. For a summary of factor analytical findings presented in this paragraph, please refer to S1 Table. Our extensive, yet not exhaustive review of the literature found that the six-factor structure of the SCS has been investigated in eleven studies and was confirmed in eight, which included college students, clinical and non-clinical samples, community samples, and the general population responding to the original SCS or translated versions of the SCS [12, 17–23]. The six-factor structure, however, was not confirmed in three studies using community, clinical, and online recruited samples [23–25]. Neff [12] proposed the existence of three conceptual SCS dimensions, but could not demonstrate them empirically. The three SCS factors corresponding to the three bipolar dimensions have also been investigated in two studies conducted with “psychologically-minded” individuals (e.g., college students) [18, 26], but the three factorial structure could not be demonstrated. A two-factor SCS structure corresponding to the positively phrased items (from self-kindness, common humanity, mindfulness subscales) and the negatively phrased items (from self-judgment, isolation, over-identification subscales), respectively, was investigated by 7 studies, and was replicated in 4 studies using college students, community and mixed samples [24, 26–28]. The two-factor SCS structure was not replicated in three studies with the general population, community members, psychologically-minded adults (e.g., university students and graduates), and a clinical sample [18, 19, 23]. In line with a possible two-factor structure of SCS, Neff [29] proposed that self-kindness, common humanity, and mindfulness represent a “self-compassionate frame-of-mind”. Similarly, Gilbert, McEwan [30] used the terms “*self-compassion*”to refer to a composite of the three positive subscales of SCS and “*self-coldness*” for the composite of the three negative subscales. A unifactorial structure of SCS has been investigated in at least 10 studies, none of which confirmed the presence of a single factor [12, 19, 20, 22, 23, 25, 26, 28, 31]. A higher order factor model with six first order factors and one second/higher order factor was confirmed in some studies [12, 21, 32, 33], but not in others [19, 20, 22–24, 27, 31]. A higher order factor model with six first order factors and two higher order factors was investigated by two studies, the model converged in one study [31], but not the other [22]. Bifactor models with six group factors and one or two general factors have been investigated in few studies: one study provided support for one general factor in three separate samples (college students, MTurk-recruited sample, meditators; MTurk of Mechanical Turk is an engine used to collect data from participants online, which is owned by Amazon), but not among a clinical sample [23]; a second study conducted with college students found support for two general factors, self-compassion and self-coldness [26]; a third study conducted with an online recruited sample reported inconclusive findings [25]. Among the 16 validation studies summarized, five used the English SCS version [12, 20, 23, 26, 28] of the SCS and 11 used SCS translations in German [18], Italian [19], Spanish [17, 31, 33], Dutch [27], Portuguese [21, 24, 31], Hungarian [25], French [32], and Japanese [22]. It is unclear whether the inconsistency of factorial results for the SCS could be attributed to translation, whether the self-compassion construct is expressed with some variability from one culture to another, or if there are other reasons for the inconsistency.

Despite a lack of clarity on the factorial validity of SCS, there is extensive interest from the research community in studying self-compassion. For example, meta-analyses exploring the relationship between a total SCS score and psychopathology [2, 34] and SCS and well-being [1, 2] found large effect sizes (range |.47| to |.54|). In addition, Muris and Petrocchi [34] assessed associations between the SCS subscale scores and measures of psychopathology and found stronger effects for the negative SCS subscales (r range = .47 to .50) than for the positive SCS subscales (r range = -.27 to -.34). These meta-analyses indicate that the use of a total SCS score could lead to inflated associations between the SCS total scale (which includes reverse-coded items tapping psychopathology features, such as isolation, self-judgment, and over-identification with negative emotions) and psychopathology measures; the use of a total score could also obscure important information, such as the unique contribution of the positive versus negative items of the SCS [34]. The use of a total summed SCS score as a measure of effect, when in fact SCS might not be unidimensional, could also negatively impact the validity of results from empirical and intervention studies. It is especially important to examine the factor structure in different translated versions of the SCS to determine whether cultural factors should be taken into consideration when assessing self-compassion. Establishing the factor structure of the SCS in different languages is an important step in improving research using this scale.

## Study objective

The main objective of this study was to investigate the factorial structure of the SCS in a large general population sample representative of the German population. We tested several models previously found in empirical research involving the SCS, but we did not have an a priori hypothesis as to which model would be a better fit for our data. The secondary objective of this study was to assess the convergent and divergent validity of the SCS. It was hypothesized that a) the positively phrased factor(s) of the SCS found to be valid would be positively associated with measures assessing positive beliefs about the self and negatively associated with measures assessing negative beliefs about the self and distress; and b) the negatively phrased factor(s) of SCS found to be valid will be negatively associated with measures assessing positive beliefs about the self and positively associated with measures assessing negative beliefs about the self and distress.

## Methods

### Participants and procedures

Data included in this study [35, 36] were extracted from a general population survey conducted in Germany in 2012. As part of this survey, a representative sample of the German general population (age 14 or above) with respect to age and gender was selected by a specialized population survey service in Germany (USUMA, Berlin). Ethics approval for the survey was obtained from the University of Leipzig Research Ethics Board (REB; Protocol # 092-12-05032012) prior to data collection. Informed consent was obtained verbally and documented by study representatives. The study also adhered to the ethical principles of the *International Code of Marketing and Social Research Practice* by the International Chamber of Commerce and the European Society for Opinion and Marketing Research. Socio-demographic information and other self-report measures were collected by trained interviewers via face-to-face interviews conducted in participants’ homes. The only criterion for inclusion in the current analysis was being at least 18 years old and having complete responses on the study measures.

### Measures

#### Self-Compassion Scale (SCS)

The SCS [12] is a 26-item self-report measure assessing self-evaluations via six proposed subscales. Three of the subscales are phrased in a positive direction: self-kindness (e.g., “*When I’m going through a very hard time*, *I give myself the caring and tenderness I need*”), common humanity (e.g., “*When I feel inadequate in some way*, *I try to remind myself that feelings of inadequacy are shared by most people*”), and mindfulness (e.g., “*When something upsets me*, *I try to keep my emotions in balance*”). The remaining three subscales are phrased in a negative direction: self-judgment (e.g., “*I’m disapproving and judgmental about my own flaws and inadequacies*”), isolation (e.g., “*When I fail at something that’s important to me*, *I tend to feel alone in my failure*”), and over-identification (e.g., “*When I’m feeling down I tend to obsess and fixate on everything that’s wrong*”). The items are scored on a 5-point Likert scale ranging from 1 (*very rarely*) to 5 (*very often*), with higher scores indicating higher levels of the construct measured. In the development sample, which consisted of undergraduate students, the internal consistency for the total scale was α = .92 and ranged from .75 to .81 for the subscales [12]. Three-week test-retest reliability for the total scale was .92 and ranged from .80 to .88 for the subscales. Validity analyses revealed positive associations with measures of social connectedness, life satisfaction, self-esteem, and emotional processing and negative associations with measures of psychological distress, self-criticism, neurotic perfectionism, and rumination [12]. The current study used the German version of the SCS [18]. Confirmatory factor analyses with the German validation sample confirmed the six subscales, which correlated as theoretically expected (in terms of directionality) with measures of self-esteem, perfectionism, neuroticism, anxiety, and depression and had good internal consistency reliability for the subscales (Cronbach’s alpha (α) ranging from 0.66 to 0.80) and test-retest reliability (coefficients ranging from 0.72 to 0.80) [18]. In the current sample, Cronbach’s α for the subscales ranged from .71 (over-identification) to .79 (self-kindness).

#### Patient Health Questionnaire-9 (PHQ-9)

The PHQ-9 [37] is a brief self-administered screening tool assessing depressive symptomatology as per the DSM-IV criteria. It asks respondents to indicate how often during the previous two weeks they were bothered by problems such as “feeling down, depressed, or hopeless”. The items are rated on a 4-point Likert scale ranging from 0 (*not at all*) to 3 (*nearly every day*), with higher scores indicating more symptoms and/or higher severity. The PHQ-9 has shown good construct and criterion validity [37–39]. While initially developed for clinical populations, the PHQ-9 can also be used as a depression screening tool among the general population [39]. In the current study, a total score computed across all nine items (α = .86) was used in analyses.

#### Generalized Anxiety Disorder screener (GAD-2)

The GAD-2 [40] is a two-item measure assessing the frequency of anxiety symptoms experienced over the previous two weeks. Two core items (“*Feeling nervous*, *anxious*, *or on edge*” and “*Not being able to stop or control worrying*”) of the GAD-7 tool [40] were retained to create an ultra-brief screening tool. Response options were rated on a 4-point Likert scale ranging from 0 (*not at all*) to 3 (*nearly every day*). The current study used the German version of the GAD-2, which was found to have comparable validity and reliability to the GAD-7 [41].

#### Core Self-Evaluations Scale (CSES)

The CSES [42] is a 12-item scale that measures four types of core self-evaluations: locus of control, neuroticism, self-efficacy, and self-esteem. Half of the items are positively worded (e.g., “I complete tasks successfully”) while the other half are phrased negatively (e.g., “Sometimes when I fail I feel worthless”) and reverse-scored for the purpose of deriving a total scale score. Answers are rated on a 5-point Likert scale ranging from 1 (*strongly disagree*) to 5 (*strongly agree*). The CSES is scored by averaging the responses, with higher scores indicating more positive core self-evaluations. The German version of the Core Self-evaluation Scale was used in the current study [43]. Confirmatory factor analysis (CFA) conducted with a German general population sample found that the positively and negatively worded items loaded into two separate factors [35]. In the current study, two composite scores corresponding to the positive (α = .85) and negative (α = .84) factors were used in analyses.

### Data analysis

#### Assessment of the factor structure of the SCS

Descriptive statistics (mean, standard deviation, range, percentage) of demographic characteristics were computed. Based on previous reports on the factor structure of the SCS, six initial models were fitted using CFA in MPlus 7 [44]: (1) a unifactorial model encompassing all 26 items; (2) a two-factor model encompassing the positively phrased items and the negatively phrased items, respectively; (3) a three-factor model with one factor incorporating items from the self-kindness and self-judgment subscales, a second factor encompassing items from the common humanity and isolation subscales, and a third factor with items from mindfulness and over-identification subscales; (4) a six-factor model corresponding to the six subscales hypothesized by Neff [2003a]; (5) a second order model testing the hierarchical relationship between six first-order factors (six subscales) and two second-order factors, i.e., SCS positive (encompassing items belonging to the self-kindness, common humanity, mindfulness subscales) and SCS negative (encompassing items belonging to the self-judgment, isolation, over-identification subscales) and (6) a six-factor model in which we arbitrarily assigned the positively phrased items into three subscales and the negatively phrased items into three separate subscales. None of the arbitrarily created scales contained more than two items from the same original SCS subscale. The item assignment procedure for the last model was included in S2 Table. The rationale for this model was to a) compare it against the six-factor model hypothesized by Neff, as we suspected that the six subscales hypothesized by Neff were not distinct from each other, based on previously reported high correlations between the positive (r > .7) and negative (r >. 8) SCS subscales [23] and b) to help us with the selection of our best fitting model versus one that is a mere artefact of our modelling procedures. It is known that model fit indices tend to improve when more factors are being modelled [45, 46].

In addition, three bifactor models were tested. In a bifactor model, the items are modeled to concurrently load into group factors (SCS subscales) and the target factor(s) (latent overall SCS factor[s]). Bifactor models evaluate the degree to which covariance among a set of item responses can be accounted for by a single general factor that reflects common variance among all scale items, as well as specific factors reflecting additional common variance among clusters of items with similar content [47, 48]. The bifactor model can provide a useful alternative to standard correlated factor models for assessing aspects of multidimensionality and can be useful for providing possible explanations when there is a lack of clarity about dimensionality. The bifactor model can also be useful for evaluating whether a unit-weighted composite score for a single latent trait can be reasonably interpreted, versus creating subscales, in the context of identifiable multidimensionality [47–49]. In the bifactor model, the general (target) factor(s) represents the broad overarching construct that is being measured, and the group factors represent more narrowly defined subdomains [47]. In bifactor model #1, the general (target) factor represented the construct of self-compassion encompassing all 26 items of the SCS, and the group factors represented the two key subdomains, the negative and positive constructs of the SCS, respectively. In bifactor model #2 (or a two-tier bifactor model), there were two general (target) factors: the positive and negative SCS constructs, and six group factors, representing the SCS subscales, three corresponding to the negative SCS construct and three corresponding to the positive SCS construct. In bifactor #3, there was one general factor (overall SCS) and six group factors (the six SCS subscales). In the bifactor models, all items were specified to load on the general factor (or factors) plus their designated group factor, and the general and specific factors were specified to be orthogonal (uncorrelated), as per assumptions of the bifactor analysis. In order to assess the contribution of the general factor and the specific factors to explaining item covariance, we calculated explained common variance (ECV), which is the ratio of variance explained by the general factor divided by variance explained by the general plus the specific factors [47]. In addition, coefficient omega, which represents the proportion of total score variance attributed to all common factors, i.e., general and target factors, was computed. Omega is a model-based reliability estimate of the multidimensionality composite total score, which is analogous to coefficient alpha [50]. Coefficient omega hierarchical (omega-h), which represents the proportion of total score variance that can be attributed to a single common factor, was also calculated for the general factor/factors. Omega-h assesses the degree to which the total scores reflect variation on the target (general) dimension [50]. Coefficient omega-h subscale (omega-hs) was also calculated for the specific group factors in order to evaluate the degree to which the subscales provided reliable information that is unique, i.e., beyond that of the general factor. Omega-hs for the group factors represents the proportion of unique variance that can be attributed to each of the group factors (subscales) after controlling for the variance of the general factor [50]. In order to assess the unidimensionality of the SCS, we used recommended cut offs of ECV > .70 - .80 and omega-h (and omega- h subscale) > .80 to identify strong general (and group) factors [47, 51].

Item responses were ordinal Likert data and were therefore modeled using the weighted least squares (WLSMV) estimator with a diagonal weight matrix, robust standard errors, and a mean- and variance-adjusted chi-square statistic with delta parameterization [44]. The negatively phrased items were reverse coded for analyses. For the 2, 3, 6-factor, and the second order models correlations among the SCS factors were specified. In the bifactor models the factors are assumed to be orthogonal, so correlations between the factors were not allowed, as per the assumptions of the bifactor anlaysis. In addition to the chi-square test, which is highly sensitive to sample size (i.e., it can lead to erroneous rejection of the model fit) [52], a combination of other indices, i.e., the Tucker-Lewis Index (TLI) [53], the Comparative Fit Index (CFI) [54] and the Root Mean Square Error of Approximation (RMSEA) [55] was used to assess the model fit. Good fitting models are indicated by a TLI and CFI ≥ 0.95 and RMSEA ≤ 0.06 [56], although a CFI and TLI of .90 or above [57] and a RMSEA of .08 or less [58] are often regarded as indicators of an adequate model fit. Modification indices were used to identify pairs of items for which the model fit would improve if the error estimates were freed to covary and for which there appeared to be theoretically justifiable shared method effects (e.g., similar content) [59]. Further, guidelines developed by Cheung and Rensvold [60] were used to determine whether there were substantive differences in model fit between the models we tested. A difference of ≤ 0.01 in the CFI between any two models was considered indicative of similar models. To select the best fitting model, we considered overall model fit indices and differences in CFI between each two models, factor loadings, inter-scale correlations, and additionally, for the bifactor models, omega coefficients.

#### Assessment of the reliability and validity of the SCS

Cronbach’s alpha was computed for the SCS factors. Convergent and divergent validity of the SCS factors were assessed through Pearson’s correlations with the PHQ-9, GAD-2, and the two subscales of the CSES. The magnitude of the bivariate correlations was interpreted following Cohen’s effect sizes, with *r* ≤ 0.10 indicating small, *r* = 0.30 indicating moderate, and *r* = 0.50 indicating large differences [61–63]. These analyses were conducted using SPSS, version 20.

## Results

### Sample description

Data were collected from 2510 people (56.5% response rate) of which 2448 (54% female, M age = 50.23, SD = 17.39, Range = 18–91) met the age restriction (≥ 18) for the current study. Socio-demographic characteristics of the sample are displayed in Table 1.

**Table 1:** Socio-demographic characteristics of the study sample (n = 2448).

Variable	N (%)
Age groups in years	
18–29	399 (16)
30–39	343 (14)
40–49	396 (16)
50–59	499 (21)
60–69	416 (17)
≥ 70	395 (16)
Relationship status	
Married and/or cohabitation	1254 (51)
Married, but separated	36 (2)
Divorced	296 (12)
Widowed	280 (11)
Single, never married	582 (24)
Education level completed	
≤ 8	976 (40)
9–11	1034 (42)
≥ 12	427 (17)
Student (ongoing)	11 (0)
Employment status	
Full time	1031 (42)
Part-time	319 (13)
Retired	728 (30)
Unemployed	101 (4)
Other	269 (11)

### Assessment of the factor structure of the SCS

Table 2 shows fit indices for all factor models. The unifactorial model and the three-factor model both showed poor fit. The two-factor model showed better, though less than ideal, fit, χ2 (298) = 6220.94, p < .001; CFI = .87; TLI = .85; RMSEA = .09. The correlation between the two latent factors was | r | = .23, p < .001 (negative items reverse coded; see Table 3). The fit indices for the six-factor model were similar to the two-factor model, χ2 (284) = 5265.15, p < .001; CFI = .89; TLI = .87; RMSEA = .09. The correlations between the three negatively phrased latent factors ranged from .79 - .93 and between the three positively phrased latent factors ranged from .76 - .81 (see Table 3). The six-factor CFA with arbitrarily assigned items revealed similar fit indices to those of the six-factor solution presented above, χ2 (284) = 5632.23, p < .001; CFI = .88; TLI = .86; RMSEA = .09. The second order model also showed less than ideal fit, χ2 (293) = 5167.83, p < .001; CFI = .89; TLI = .88; RMSEA = .08.

**Table 2:** Summary of models tested via confirmatory factor analysis (CFA) with the items of the Self-Compassion Scale.

Model	CFA with unfreed error covariances					CFA with three^a^ freed error covariances				
	χ^2^	df	CFI	TLI	RMSEA [90% CI]	χ^2^	df	CFI	TLI	RMSEA [90% CI]
1-factor model	21128.36*	299	.53	.48	.17 [.17; .17]	19835.37*	296	.56	.51	.16 [.16; .17]
2-factor model ^b^	6220.94*	298	.87	.85	.09 [.09; .09]	5444.59*	295	.88	.87	.08 [.08; .09]
3-factor model ^c^	20780.66*	296	.53	.49	.17 [.17; .17]	19754.90*	293	.56	.51	.17 [.16; .17]
6-factor model ^d^	5265.15*	284	.89	.87	.09 [.08; .09]	4830.87*	281	.90	.88	.08 [.08; .08]
6-factor model ^e^ (Arbitrary items)	5632.23*	284	.88	.86	.09 [.09; .09]	4880.68*	281	.90	.88	.08 [.08; .08]
2^nd^ order model ^f^:	5167.83*	293	.89	.88	.08 [.08; .08]	4784.97*	290	.90	.89	.08 [.08; .08]
Bifactor model #1^g^	5052.65*	274	.89	.87	.08 [.08; .09]	-	-	-	-	-
Bifactor model #2 ^h^	4501.37*	275	.90	.89	.08 [.08; .08]	-	-	-	-	-
Bifactor model #3 ^i^	16194.59*	274	.64	.57	.15 [.15; .16]	-	-	-	-	-

*Note*. df = degree of freedom; CFI = Comparative Fit Index; TLI = Tucker-Lewis Index; RMSEA = root mean square error of approximation.

^a^ Pairs of errors for which covariances were freed: 10 and 7; 18 and 13; 19 and 5;

^b^ model with all negative items (pertaining to subscales self-judgment [SJ], isolation [IL], and over-identification (OI]) loading into SCS negative factor and all positive items (pertaining to subscales self-kindness [SK], common humanity [CH], and mindfulness (MF]) loading into SCS positive factor;

^c^ model with SK and SJ items loading into factor 1, CH and IL loading into factor 2; MF and OI loading into factor 3;

^d^ model with the six factors corresponding to the six subscales (SK, SJ, CH, IL, MF, OI);

^e^ model with six factors to which items were arbitrarily assigned (see details in S 2);

^f^ model with six first order factors (SK, SJ, CH, IL, MF, OI) and two second order factors (SCS positive and SCS negative);

^g^ model with one general factor (self-compassion general construct) and two group factors (SCS positive and SCS negative);

^h^ two-tier bifactor model with two general factors (SCS positive and SCS negative) and six group factors, three per the SCS positive dimension (SK, SJ, CH) and three for the SCS negative dimension (IL, MF, OI);

^i^ model with one general factor (SCS) and six group factors (SK, SJ, CH, IL, MF, OI)

*p < .001.

**Table 3:** Additional results for the bifactor models, including omega coefficients and ECV computations.

Model	Omega (ω)	Omega-h (ω_h_)	ECV	Omega-hs (ω_hs)_
Bifactor model #1^a^	.94	.42	.33	.83 SCS positive
				.43 SCS negative
Bifactor model #2^b^				
SCS positive general factor	.91	.83	.72	.18 SK; .23 CH; .13 MF
SCS negative general factor	.92	.86	.77	.23 SJ; .16 IL; .01 OI
Bifactor model #3^c^	-	-	-	-

*Note*. SCS = self compassion scale

^a^ model with one general factor (self-compassion general construct) and two group factors (SCS positive and SCS negative);

^b^ model with two general factors (SCS positive and SCS negative) and six group factors (SK, SJ, CH, IL, MF, OI);

^c^ model with one general factor (SCS) and six group factors (SK, SJ, CH, IL, MF, OI)

Omega = proportion of total score variance attributed to all common factors, i.e., general and target factors;

Omega-h (omega hierarchical) = proportion of total score variance that can be attributed to a single common factor, calculated for the general factor/factors;

Omega-hs (omega hierarchical subscale) = proportion of unique variance that can be attributed to each of the group factors after controlling for the variance of the general factor;

ECV (explained common variance) = the ratio of variance explained by the general factor divided by variance explained by the general plus the specific factors (or proportion of common variance due to the general factor).

Upon inspecting the Modification Indices of the six-factor solution, three pairs of item errors were allowed to freely covary: items 10 and 7 (common humanity); items 18 and 13 (isolation); and items 19 and 5 (self-kindness), which resulted in minor improvements of the model fit for the two-factor model and the six-factor model (Table 2). The second order model did not further improve by freeing these error terms. Given the differences in CFI close to 0.01, the two-factor model, the six factor model, and the second order model were considered as having comparable model fit. For comparison purposes, the results of the CFAs with and without freeing covariances were included in Table 2.

The bifactor model #1, with one general (target) factor and two group factors, showed less than ideal fit, χ2 (274) = 5052, p < .001; CFI = .89; TLI = .87; RMSEA = .08. The omega coefficient (ω) was .94 and omega-hierarchical (ω_h_) for the general factor was .42. The explained common variance for the common factor (ECV) was .33, and omega-h subscale (ω_hs)_ was .83 for the SCS positive group factor and .43 for the SCS negative group factor.

The bifactor model #2, the two-tier bifactor model with two general (target) factors and six group factors, showed adequate fit, χ2 (275) = 4501.37, p < .001; CFI = .90; TLI = .89; RMSEA = .08). For the SCS positive general factor ω = .91, ω_h_ for the positive factor = .83, and ECV = .72; ω_hs_ = .18 for self-kindness, ω_hs_ = .23 for common humanity, and ω_hs_ = .13 for mindfulness positive group factors. For the SCS negative general factor ω = .92, ω_h_ for the negative factor = .86, and ECV = .77; ω_hs_ = .23 for self-judgment, ω_hs_ = .16 for isolation, and ω_hs_ = .01 for over-identification negative group factors. The omega coefficients and the ECV computations for the bifactor models (1 and 2) were included in Table 3.

The bifactor model #3, with one general factor and six group factors, yielded a poor fit, χ2 (274) = 16194.59, p < .001; CFI = .64; TLI = .57; RMSEA = .15. Given the poor model fit, omega coefficients were not computed for this model.

### Validity and reliability of the SCS factors

#### SCS latent factor scores computed in MPlus

In Mplus, we modelled latent factors. Correlations among the latent factors pertaining to the six-factor and two-factor solutions, and computed in Mplus, were included in Table 4. Factor loadings for the two-factor CFA were included in Table 5 and the loadings for the six-factor CFA were included in S3 Table. In the two-factor solution, there was a moderate positive correlation between the SCS positive factor and the SCS negative factor. In the six factor-solution, there were large positive correlations between the three positive and the three negative latent factors, respectively.

**Table 4:** Correlations between the study measures and the six SCS factors and the two SCS factors.

Measure	SK	CH	MF	POS	SJ	IL	OI	NEG	PHQ-9	GAD-2	CSES Positive	CSES Negative
SCS self-kindness (SK)	1^(^^a^^/^^b^^)^	.60**	.62**	.86**	.11**	.09**	.16**	.12**	-.08**	-.06*	.22**	-.06*
SCS common humanity (CH)	.77	1^(^^a^^/^^b^^)^	.63**	.86**	.21**	.16**	.23**	.21**	-.01	.00	.14**	.00
SCS mindfulness (MF)	.76	.81	1^(^^a^^/^^b^^)^	.86**	.25**	.06*	.16**	.16**	-.07**	-.04	.30**	-.11**
**SCS positive factor (POS)**	-	-	-	1^(^^a^^/^^b^^)^	.24**	.14**	.23**	.21**	-.05	-.02	.24**	-.04
SCS self-judgment (SJ)	.15	.29	.32	-	1^(^^a^^/^^b^^)^	.56**	.65**	.83**	.35**	.30**	-.19**	.40**
SCS isolation (IL)	.15	.23	.06	-	.79	1^(^^a^^/^^b^^)^	.68**	.87**	.43**	.37**	-.37**	.59**
SCS over-identification (OI)	.22	.32	.19	-	.93	.93	1^(^^a^^/^^b^^)^	.89**	.38**	.34**	-.27**	.58**
**SCS negative factor (NEG)**	-	-	-	.23	-	-	-	1^(^^a^^/^^b^^)^	.45**	.40**	-.34**	.65**

*Note*. *N*’s ranged from 2426 to 2446 due to missing data. The items of the SJ, Il, and OI subscales were reverse coded.

SCS = Self-Compassion Scale; PHQ-9 = Patient Health Questionnaire-9; GAD-2 = Generalized Anxiety Disorder-2; CSES Positive = Core Self Evaluations Scale: Positive Composite Score; CSES Negative = Core Self Evaluations Scale: Negative Composite Score.

^a^ Above the diagonal: Correlations between summed subscale scores computed in SPSS;

^b^ Below the diagonal: Correlations between latent factors computed in MPlus.

Bonferroni corrected

*p < .006.

**p < .001.

**Table 5:** Factor loadings and Confidence Intervals (CI) for the two-factor structure of the Self-Compassion Scale (SCS).

Item	Factor loading	95% CI
**SCS Positive Latent Factor**		
3. When things are going badly for me, I see the difficulties as part of life that everyone goes through.	0.63	[0.60, 0.66]
5. I try to be loving towards myself when I’m feeling emotional pain.	0.61	[0.58, 0.64]
7. When I'm down and out, I remind myself that there are lots of other people in the world feeling like I am.	0.58	[0.55, 0.61]
9. When something upsets me I try to keep my emotions in balance.	0.69	[0.66, 0.71]
10. When I feel inadequate in some way, I try to remind myself that feelings of inadequacy are shared by most people.	0.61	[0.59, 0.64]
12. When I’m going through a very hard time, I give myself the caring and tenderness I need.	0.72	[0.70, 0.74]
14. When something painful happens I try to take a balanced view of the situation.	0.70	[0.68, 0.72]
15. I try to see my failings as part of the human condition.	0.63	[0.61, 0.66]
17. When I fail at something important to me I try to keep things in perspective.	0.66	[0.64, 0.69]
19. I’m kind to myself when I’m experiencing suffering.	0.61	[0.58, 0.63]
22. When I'm feeling down I try to approach my feelings with curiosity and openness.	0.63	[0.61, 0.65]
23. I’m tolerant of my own flaws and inadequacies.	0.52	[0.49, 0.55]
26. I try to be understanding and patient towards those aspects of my personality I don't like.	0.71	[0.69, 0.73]
**SCS Negative Latent Factor**		
1. I’m disapproving and judgmental about my own flaws and inadequacies.	0.57	[0.55, 0.60]
2. When I’m feeling down I tend to obsess and fixate on everything that’s wrong.	0.71	[0.69, 0.73]
4. When I think about my inadequacies, it tends to make me feel more separate and cut off from the rest of the world.	0.71	[0.68, 0.73]
6. When I fail at something important to me I become consumed by feelings of inadequacy.	0.73	[0.70, 0.75]
8. When times are really difficult, I tend to be tough on myself.	0.52	[0.49, 0.55]
11. I’m intolerant and impatient towards those aspects of my personality I don't like.	0.62	[0.59, 0.65]
13. When I’m feeling down, I tend to feel like most other people are probably happier than I am.	0.61	[0.58, 0.63]
16. When I see aspects of myself that I don’t like, I get down on myself.	0.74	[0.72, 0.76]
18. When I’m really struggling, I tend to feel like other people must be having an easier time of it.	0.60	[0.57, 0.63]
20. When something upsets me I get carried away with my feelings.	0.60	[0.57, 0.63]
21. I can be a bit cold-hearted towards myself when I'm experiencing suffering.	0.60	[0.58, 0.63]
24. When something painful happens I tend to blow the incident out of proportion.	0.71	[0.69, 0.73]
25. When I fail at something that's important to me, I tend to feel alone in my failure.	0.57	[0.55, 0.60]

#### SCS subscale scores computed in SPSS

In SPSS, we computed summed subscale scores. Correlations between the study measures and the six original SCS subscales and the two SCS subscales (derived from all of the positive items and all of the negative items, respectively), computed in SPSS, were included in Table 3. There was a moderate positive correlation between the SCS positive and SCS negative subscales. There were large positive correlations (.60 < *r* < .63) among the three positively phrased subscales, self-kindness, common, humanity, and mindfulness). There were also large positive correlations (.56 < *r* < .68) among the three negatively phrased subscales, self-judgment, isolation, and over-identification. Further, there were large positive correlations (*r* > .85) between the three positively phrased SCS subscales. Similarly, there were large positive correlations between the negatively phrased SCS subscales (*r* > .80). The associations between the positive SCS subscales and the PHQ-9, the GAD-2, and the CSES negative were negligible, but there was a small positive association with the CSES positive. The negative SCS factors showed moderate-to-large correlations with the PHQ-9, the GAD-2, and the CSES negative, and small to moderate negative correlations with the CSES positive.

#### Reliability assessment

In the six-factor model, Cronbach’s alphas for the SCS subscales were .79 for self-kindness, .73 for common humanity, .77 for mindfulness, .73 for self-judgment, .77 for isolation and .71 for over-identification. In the two-factor SCS model, alpha was .88 for the SCS positive subscale and .87 for the SCS negative subscale.

## Discussion

Initial CFA showed acceptable and comparable model fit indices among the two- and six-factor models and for the second order model. A six-factor CFA with arbitrarily assigned items revealed a model fit almost identical to the fit of the six-factor model using Neff’s original item assignment, which renders little meaning to the computation of six subscales scores. The second order and six-factor models did not improve fit to the data substantively over the two-factor model [60]. Further, bifactor models supported the presence of two latent SCS factors. The first bifactor model that we tested with one general factor (SCS) and two group factors (SCS positive and SCS negative) had a large omega value, but much lower omega-h and ECV values. This shows that little of the variance in SCS total scores is due to the general factor and, conversely, that a considerable proportion of the variance in total scores is due to the two group factors, i.e., SCS positive and SCS negative. Further, omega-h group coefficients showed a high value for the SCS positive factor and much lower value for the SCS negative factor. This suggests that the SCS positive factor captures most of the variance in the SCS construct raising questions about the relevancy of the negative factor to the overall SCS construct. The second bifactor model we tested, with two general factors and six group factors, found high omega and omega-h coefficients and high ECV values for the general factors, and virtually nil omega-h group coefficients for the six group factors. The omega-h group coefficients, which are indicators of reliability of additional variance (i.e., beyond the variance explained by the two general factors) provided evidence against the six factors, suggesting that the six factors do not reliably explain additional variance beyond the two general factors, SCS positive and SCS negative.

In the six-factor model, the magnitude of the correlations among the positive latent factors and among the negative latent factors suggested significant redundancy within each set. We reported both correlations between SCS latent factors, computed as part of the CFA in MPlus (range .76 - .93) and correlations between SCS subscale scores, computed in SPSS (range .60 - .68). Error in the measurement reduces the correlation between variables by definition, as it is unrelated to the construct being measured. Therefore the SPSS correlations are lower than the MPlus correlations, while the latter are more accurately describing the relationship between the constructs. Given the magnitude of the correlations within each set, positive and negative, it seems reasonable to suggest that the sub-constructs within each set overlap so much that it is difficult to distinguish between them [64]. Further, the correlation matrix showed that the positively phrased SCS subscales (self-kindness, common, humanity, and mindfulness) and the SCS positive subscale showed a similar pattern of associations with the other study measures. In the same vein, the three negatively phrased SCS subscales (self-judgment, isolation, and over-identification) and the SCS negative subscale also showed a similar pattern of associations with the study measures. These patterns of associations did not suggest an advantage of using the six SCS subscales over the two SCS subscales, i.e., SCS positive and SCS negative.

Given all of these considerations, the two-factor solution, with SCS positive and SCS negative latent factors, was deemed to be the best fit for our observed data. The two-factor solution we identified in this study is consistent with previous factor-analytic studies, which also found a two-factor structure for SCS [24, 26–28], and with theoretical stances that the positive subscale and the negative subscale of SCS are inherently different from each other [28–30]. While the two SCS factors that we identified seem robust, the convergent and divergent analyses paint an unclear picture. Opposite to our hypothesis, which was informed by literature reviews on the relationship between self-compassion and well-being as well as psychopathology, we found that the SCS positive factor was unrelated to distress and only minimally related to positive self-evaluations. Future research is needed to determine if this unexpected finding is replicated and to generate an explanation for it. In agreement with our hypothesis, the SCS negative factor was consistently associated with measures of distress and negative self-evaluations. While it is premature to make definite claims about the positive SCS factor given the limited number of measures included in the study, it seems reasonable to suggest that the SCS negative factor assesses tendencies towards self-criticism, over-identification with negative self-statements, and an overall negative cognitive style, all of which represent symptoms of depression [24, 26, 27, 65] and/or maladaptive interpersonal styles, such as neuroticism [28, 34].

A strength of the present study was the large, representative sample of the general population and the comprehensive modelling approach. There are some limitations to be considered when interpreting the results of this study. First, this study used a German translation of the SCS. Therefore it is unclear whether the results are generalizable to other languages or cultures. Second, this study used a general population sample, which is arguably different from a more psychologically-minded sample, such as social science students or users of psychology-related websites, on which the English SCS was first developed and tested and which were used to initially validate the German SCS. The language and population considerations could have affected the model fit indices we found in the current study, which were less than ideal, but overall they provided a reasonable (adequate) fit for our data. Furthermore, given the limited number of measures for convergent and divergent validity testing included in this study, particularly the lack of measures assessing positive constructs (e.g., optimism, hopefulness, quality of life), it is difficult to demonstrate the construct validity of the SCS positive and negative factors. Future research is needed to elucidate whether the negative items of the SCS capture aspects that are unique to self-compassion or whether they tap vulnerabilities to depression (e.g., self-criticism) and/or other psychopathology [34]. Future research is also needed to cross-validate our results in more culturally and linguistically heterogeneous samples. Future exploratory research could use exploratory techniques such as exploratory structural equation modeling (ESEM) to further elucidate the structure of the SCS scale across various populations.

In sum, this study using data collected in Germany found that three models of SCS produced similar fit indices, with no substantive difference: a two-factor model, a six-factor model, and a hierarchical model with six first-order factors and two second (higher) order factors. Notably, the six-factor model hypothesized by Neff produced similar fit indices to a six-factor model with positive and negative items arbitrarily assigned to three positive and three negative factors [12]. The bifactor models supported a two-factor solution. The one-factor and three-factor models did not converge. While six factors can be modeled, the three negative factors and the three positive factors, respectively, do not seem to reflect reliable or meaningful variance beyond one positive and one negative item factor. As such, we recommend the use of two subscale scores to capture a positive factor and a negative factor when administering the German SCS to general population samples and we strongly advise against the use of a total score across all SCS items.

## Supporting information

S1 Table Summary of results from factor analytic studies assessing the structure of the Self-Compassion Scale. (PDF)

S2 Table Distribution of the Self-Compassion Scale items to three positive arbitrary and three negative arbitrary factors. (PDF)

S3 Table Factor loadings and Confidence Intervals (CI) for the six-factor structure of the Self-Compassion Scale. (PDF)
