# Supplementary material for: Structural validation of the Self-Compassion Scale with a German general population sample
Source: PLoS One. 2018 Feb 6;13(2):e0190771. doi: 10.1371/journal.pone.0190771 (PMC5800544; doi:10.1371/journal.pone.0190771)
Supplement: S1 Table — (PDF) [file pone.0190771.s001.pdf]

S 1: Summary of results from factor analytic studies assessing the structure of the Self-Compassion Scale

Study	Sample/size	Language	Analysis	Factors Tested	Reported Model fit Indices	Reported Model Fit Qualifiers^{+}	Reported Criteria for Adequate Model Fit
Neff, 2003 [12]	University students (n=391)	English	CFA	1F	CFI=.84, NNFI=.80 (self-kindness and self-judgment items)	Poor fit	Not reported
				1F	CFI=.59, NNFI=.43 (common humanity and isolation items)	Poor fit	
				1F	CFI=.83, NNFI=.75 (mindfulness and over-identification items)	Poor fit	
				2F	CFI=.91, NNFI=.88 (self-kindness/self-judgment)	Adequate fit	
				2F	CFI=.99, NNFI=.99 (common humanity /isolation)	Adequate fit	
				2F	CFI=.96, NNFI=.94 (mindfulness/over-identification)	Adequate fit	
				6F	CFI=.91, NNFI=.90	Adequate fit	
				Second order (6>1)	CFI=.90, NNFI=.88	Acceptable fit	
Neff, 2017 [23]	Students (n=222)	English	CFA	1F	RMSEA=.09, SRMR=.08, CFI=.79, TLI=.77, AIC=14438.04	Poor fit	CFI, TLI ≥ .90;
				2F	RMSEA=.07, SRMR=.06, CFI=.88, TLI=.87, AIC=14191.63	Poor fit	RMSEA, SRMR ≤
				6F	RMSEA=.05, SRMR=.05, CFI=.93, TLI=.92, AIC=14047.81	Adequate fit	.10, AIC
				Second order (6>1)	RMSEA=.07, SRMR=.07, CFI=.89, TLI=.88, AIC=.14153.43	Poor fit	
				Bifactor (6>1)	RMSEA=.06, SRMR=.06, CFI=.91, TLI=.89, AIC=14098.65, ω= .95, ωH=.90	Adequate fit	Omega and omega-
							h coefficients, for
	MTurk-recruited sample (N=1394)			1F	RMSEA=.10, SRMR=.08, CFI=.74, TLI=.72, AIC=100063.94	Poor fit	bifactor models
				2F	RMSEA=.07, SRMR=.05, CFI=.88, TLI=.87, AIC=97446.58	Poor fit	[cutoff not
				6F	RMSEA=.05, SRMR=.04, CFI=.94, TLI=.93, AIC=96229.10	Adequate fit	reported]
				Second order (6>1)	RMSEA=.06, SRMR=.08, CFI=.89 TLI=.88, AIC=97214.10	Poor fit	
				Bifactor (6>1)	RMSEA=.06, SRMR=.07, CFI=.91, TLI=.89, AIC=96823.54, ω= .95, ωH=.89	Adequate fit	
	Meditators (N=215)			1F	RMSEA=.11, SRMR=.09, CFI=.74, TLI=.72, AIC=12651.00	Poor fit	
				2F	RMSEA=.08, SRMR=.06, CFI=.87, TLI=.86, AIC=12258.42	Poor fit	
				6F	RMSEA=.06, SRMR=.06, CFI=.93, TLI=.92, AIC=12071.63	Adequate fit	
				Second order (6>1)	RMSEA=.07, SRMR=.08, CFI=.89, TLI=.88, AIC=12190.95	Poor fit	
				Bifactor (6>1)	RMSEA=.07, SRMR=.07, CFI=.91, TLI=.90, AIC=12115.61, ω= .96, ωH=.90	Adequate fit	
	Clinical sample (N=390)			1F		Poor fit	
				2F	RMSEA=.11, SRMR=.10, CFI=.64, TLI=.61, AIC=28263.28	Poor fit	
				6F	RMSEA=.08, SRMR=.07, CFI=.83, TLI=.82, AIC=27495.59	Poor fit	
				Second order (6>1)	RMSEA=.07, SRMR=.06, CFI=.88, TLI=.87, AIC=27307.50	Poor fit	
				Bifactor (6>1)	RMSEA=.09, SRMR=.10, CFI=.80, TLI=.78, AIC=27639.30	Poor fit	
					RMSEA=.08, SRMR=.09, CFI=.84, TLI=.81, AIC=27474.55, ω= .94, ωH=.85		

Hupfeld, 2011 [18]	Psychologically minded adults (n=561)	German	ESEM	2F	X^2=1154.91, p<.01, RMSEA=.08, SRMR=.06, CFI=.82	Poor fit	Not reported, but references provided [54]
				3F	X^2=835.45, p<.01, RMSEA=.07, SRMR=.05, CFI=.88	Acceptable fit	
				6F	X^2=280.98, p<.01, RMSEA=.031, SRMR=.019, CFI=.98	Adequate fit	
			CFA	2F	Not reported	Poor	
				3F	Not reported	Poor	Not reported, but references provided [54]
				6F	X^2=961.73, p<.01, RMSEA=.065, SRMR=.064, CFI=.86	Acceptable fit	
				Second order (6>1)	X^2=1258.56, p<.01, RMSEA=.077, SRMR=.081, CFI=.83	Acceptable fit	
Petrocchi, 2014 [19]	Adults recruited online (n=424; 40% students)	Italian	CFA	1F	Not reported	Poor fit	CFI, TLI ≥ .90; 0.5 ≤ RMSEA ≤ .08 SRMR ≤ .08
				2F	Not reported	Poor fit	
				6F	X^2=320.28, p<.001, RMSEA=.08, SRMR=.07, TLI=.97, CFI=.90	Adequate fit	
				Second order (6>1)	Not reported	Poor fit	
Garcia-Campayo [17]	College (health science) students (n=268)	Spanish	CFA	6F	X^2= not reported; RMSEA=.06, SRMR=.05; CFI=.95; GFI=.93	Adequate fit	CFI, GFI > .90; SRMR, RMSEA < .08
Williams, 2014 [20]	Convenience sample (n=821)	English/ UK	CFA	1F	X^2=3937.49, p<.001, RMSEA=.12, SRMR=.10, CFI=.68, NNFI=.65	Poor fit	Good (and adequate) criteria for adequate fit CFI, NFFI ≥ .95 (≥ .90); RMSEA ≤ .06 (≤ .10); SRMR ≤ .05 (≤ .10)
				6F	X^2=1472.68, p<.001, RMSEA=.07, SRMR=.06, CFI=.90, NNFI=.88	Adequate fit	
				Second order (6>1)	X^2=2142.74, p<.001, RMSEA=.09, SRMR=.09, CFI=.84, NNFI=.81	Poor fit	
	Adults practicing meditation (n=211)			1F	X^2=4649.68, p<.001, RMSEA=.13, SRMR=.10, CFI=.64, NNFI=.61	Poor fit	
				6F	X^2=2629.61, p<.001, RMSEA=.10, SRMR=.08, CFI=.80, NNFI=.77	Acceptable	
				Second order (6>1)	X^2=3104.76, p<.001, RMSEA=.11, SRMR=.10, CFI=.77, NNFI=.73	Poor fit	
	Adults with major depressive disorder (n=390)			1F	X^2=3837.89, p<.001, RMSEA=.12, SRMR=.10, CFI=.63, NNFI=.59	Poor fit	
				6F	X^2=1673.59, p<.001, RMSEA=.08, SRMR=.06, CFI=.85, NNFI=.83	Acceptable fit	
				Second order (6>1)	X^2=2450.86, p<.001, RMSEA=.09, SRMR=.10, CFI=.77, NNFI=.74	Poor fit	
Lopez, 2015 [27]	Community sample (n=1643)	Dutch	EFA	2F	No model testing	N/a	N/a
			CFA	6F	X^2=3779.47, p<.001, RMSEA=.10, WRMR=3.15, CFI= 0.90, TLI=.88	Poor fit	Good (and adequate) fit: CFI, TLI ≥ .95 (≥ .90); RMSEA ≤ .06 (≤ .08); WRMR ≤ 1.0
			CFA	Second order (6>1)	Not reported	Poor fit	

Costa, 2015 [24]	Mixed community and clinical sample (n=220)	Portuguese	CFA	2F<br>6F<br>Second order (6>1)	X^2=738.88, p<.000, RMSEA=.07, TLI=.87, CFI=.88<br>X^2=597.60, p<.000, RMSEA=.07, TLI=.87, CFI=.88<br>X^2=885.58, p<.000, RMSEA=.10, TLI=.75, CFI=.78	Acceptable fit<br>Poor fit<br>Poor fit	CFI, TLI ≥ .90; RMSEA ≤ .06
Castilho, 2015 [21]	Mixed clinical sample (n=316)	Portuguese	CFA	6F<br>Second order (6>1)	X^2/df = 2.37, p<.001, RMSEA=.07, TLI=.88, CFI=.90<br>X^2/df = 2.98, p<.001, RMSEA=.08, TLI=.91, CFI=.94	Adequate fit<br>Adequate fit	X^2/df > 2; CFI, TLI ≥ .90; RMSEA ≤ .10
	Mixed non-clinical sample (n=1128)			6F<br>Second order (6>1)	X^2/df = 5.91, p<.001, RMSEA=.07, TLI=.91, CFI=.92<br>X^2/df = 5.98, p<.001, RMSEA=.07, TLI=.91, CFI=.92	Adequate fit<br>Adequate fit	
Arimitsu, 2014 [22]	General population (n=366)	Japanese	CFA	1F<br>6F<br>Second order (6>1)<br>Second order (6>2)	X^2/df = 7.31, RMSEA=.13, CFI=.40, NNFI=.35<br>X^2/df = 2.61, RMSEA=.07, CFI=.86, NNFI=.83<br>X^2/df = 3.68, RMSEA=.09, CFI=.75, NNFI=.73<br>X^2/df = 2.68, RMSEA=.07, CFI=.85, NNFI=.83	Poor fit<br>Poor fit<br>Poor fit<br>Poor fit	CFI ≥ .95, RMSEA ≤ .06
Pfattheicher, 2017 [28]	Convenience sample (n=576)	English	CFA	1F<br>2F<br>Second order (6>1)<br>Second order (6>2)<br>6F	X^2=2645, p<.001, RMSEA=.12, CFI=.77, PCFI=.65<br>X^2=1445, p<.001, RMSEA=.08, CFI=.89, PCFI=.75<br>X^2=1300, p<.001, RMSEA=.08, CFI=.90, PCFI=.75<br>X^2=959, p<.001, RMSEA=.06, CFI=.93, PCFI=.78<br>X^2=826, p<.001, RMSEA=.06, CFI=.95, PCFI=.77	Poor fit<br>Poor fit<br>Poor fit<br>Adequate fit<br>Adequate fit	CFI > .90, PCFI [cut off value not specified], RMSEA < .08,
			SEM	6F > 2F	X^2=958.55, RMSEA=.06, CFI=.93, ωPositive factor=.95; ωNegative factor=.93	Adequate fit	
Brenner, 2017 [26]	College students (n=1115)	English	CFA	1F<br>2F<br>3F<br>6F<br>Second order (6>1)<br>Second order (6>2)<br>Bifactor (6>1)<br>Bifactor (6>2)[e]	X^2=6396.59, RMSEA=.14, SRMR=.10, CFI=.85, NNFI=.81, AIC= 6500.59<br>X^2=2070.34, RMSEA=.07, SRMR=.06, CFI=.96, NNFI=.94, AIC= 2176.34<br>X^2=6552.39, RMSEA=.14, SRMR=.10, CFI=.84, NNFI=.81, AIC= 6662.39<br>X^2=1332.52, RMSEA=.06, SRMR=.05, CFI=.97, NNFI=.96, AIC= 1466.52<br>X^2=2156.57, RMSEA=.08, SRMR=.10, CFI=.95, NNFI=.94, AIC= 2272.57<br>X^2=1537.59, RMSEA=.06, SRMR=.05, CFI=.97, NNFI=.95, AIC= 1659.59<br>X^2=1995.72, RMSEA=.08, SRMR=.13, CFI=.96, NNFI=.94, AIC= 2151.72<br>X^2=1244.36, RMSEA=.06, SRMR=.17, CFI=.98, NNFI=.96, AIC= 1400.36<br>- General Self compassion (ωH=.84, PRV=.92, ECV=.75); SK (ωHS=.18, PRV=.21); CH (ωHS=.27, PRV=.35); MF (ωHS=.02, PRV=.03);<br>- General Self coldness (ωH=.88, PRV=.94, ECV=.68); SJ (ωHS=.12, PRV=.15); IL (ωHS=.12, PRV=.15); OI (ωHS=.18, PRV=.20);	Poor fit<br>Adequate fit<br>Poor fit<br>Adequate fit<br>Adequate fit<br>Adequate fit<br>Adequate fit<br>Adequate fit	CFI, NNFI ≥ .95, SRMR ≤ .08, and RMSEA ≤ .06.<br>6 < Δ AIC > 10<br><br>ωH, ωHS >.80, PRV > .75, ECV > .60
Tóth-Király, 2016 [25]	Online sample (n=505)	Hungarian	CFA	6F<br>Bifactor (6>1)	X^2=1755.66, RMSEA=.10, CFI=.85, TLI=.82<br>X^2=3025.58, RMSEA=.14, CFI=.71, TLI=.66	Poor fit<br>Poor fit	Good (and adequate) fit: CFI, TLI ≥.95 (≥.90), RMSEA≤06 (≤0.08)
			ESEM	6F<br>Bifactor (6>1)	X^2=509.92, RMSEA=.06, CFI=.97; TLI=.94<br>X^2= 482.85, RMSEA=.06, CFI=.97, TLI=.95,	Adequate fit<br>Adequate fit,	

					- General Self compassion (ω=.88, ωH=.53); Subscales (ωHS, SK=.53, CH=.72, MF=.09, SJ=.45, IS=.24, OI=.17)	Good fit	ΔCFI ≤0.010, ΔTLI ≤0.010, and ΔRMSEA ≤0.015.
Kotsou, 2016 [32]	Online sample (n=1554)	French	CFA	6F<br>Second order (6>1)<br>Bifactor (6>1)	X^2=1372, p<.001, X^2/df =4.95, RMSEA=.05, SRMR=.04, CFI=.94, TLI=.93<br>X^2=2284, p<.001, X^2/df =7.98, RMSEA=.07, SRMR=.07, CFI=.88, TLI=.89<br>X^2=1815, p<.001, X^2/df =6.23, RMSEA=.06, SRMR=.05, CFI=.92, TLI=.91, ω=.94	Good fit<br>Good fit<br>Good fit	Good (and adequate) fit: CFI, TLI > .90; 0 ≤ SRMR ≤ .05 (.05 ≤ SRMR ≤ .10) 0 ≤ RMSEA ≤.05. (.05 ≤ RMSEA ≤ .08
deSouza, 2016 [33]	Online sample (n=432)	Brasilian Portuguese	CFA	6F<br>Second order (6>1)<br>Bifactor (6>1)	X^2=895.90, p<.001, RMSEA=.07, CFI=.94, TLI=.93<br>X^2=1407.49, p<.001, RMSEA=.09, CFI=.89, TLI=.87<br>X^2=1203.88, p<.001, RMSEA=.09, CFI=.90, TLI=.89	Good fit<br>Adequate fit<br>Adequate fit	CFI, TLI ≥ .90 RMSEA [not reported]
Montero-Marín, 2016 [31]	Primary care professionals (Brazil: n=406) (Spain: n=414)	Brazilian Portuguese<br><br>Spanish	CFA Brazilian (Spanish) samples	1F	X^2=2266.31, p<.001, X^2/df =7.58, RMSEA=.13, SRMR=.13, CFI=.58, AIC=2370.31<br>(X^2=2164.46, p<.001, X^2/df =7.24, RMSEA=.12, SRMR=.14, CFI=.55, AIC=2268.46)	Poor fit<br>Poor fit	Good (and adequate) fit: X^2/df <3 (X^2/df <5)
				2F	X^2=675.07, p<.001, X^2/df =2.27, RMSEA=.05, SRMR=.07, CFI=.84, AIC=1179.86<br>(X^2=683.28, p<.001, X^2/df =2.29, RMSEA=.05, SRMR=.09, CFI=.79, AIC=1250.93)	Poor fit<br>Poor fit	CFI ≥.90 RMSEA ≤.06 SRMR < .08<br><br>lowest AIC
				6F	X^2=589.14, p<.001, X^2/df =2.07, RMSEA=.05, SRMR=.06, CFI=.89, AIC=947.65<br>(X^2=582.14, p<.001, X^2/df =2.05, RMSEA=.05, SRMR=.08, CFI=.86, AIC=993.42)	Adequate fit<br>Adequate fit	
				Second order (6>1)	X^2=699.29, p<.001, X^2/df =2.39, RMSEA=.06, SRMR=.14, CFI=.78, AIC=1439.11<br>(X^2=692.16, p<.001, X^2/df =2.36, RMSEA=.06, SRMR=.14, CFI=.76, AIC=1385.50)	Poor fit<br>Poor fit	
				Second order (6>2)	X^2=858.12, p<.001, X^2/df =2.94, RMSEA=.07, SRMR=.07, CFI=.88, AIC=1067.87<br>(X^2=949.87, p<.001, X^2/df =3.25, RMSEA=.07, SRMR=.09, CFI=.84, AIC=1067.87)	Acceptable<br>Poor fit	
				Second order	X^2=1237.71, p<.001, X^2/df =4.27, RMSEA=.09, SRMR=.12, CFI=.80,	Poor fit	

	(6>3[pos])	AIC=1359.71<br>(X^2=1261.98, p<.001, X^2/df =4.35, RMSEA=.09, SRMR=.13, CFI=.76, AIC=1383.98)	 Poor fit
	3F [pos]	X^2=244.04, p<.001, X^2/df =3.94, RMSEA=.09, SRMR=.05, CFI=.91, AIC=302.04<br>(X^2=285.73, p<.001, X^2/df =4.61, RMSEA=.09, SRMR=.06, CFI=.89, AIC=343.73)	Good fit<br>Good fit
	Second order (3[pos]>1 [SCS pos])	X^2=244.04, p<.001, X^2/df =3.94, RMSEA=.09, SRMR=.05, CFI=.91, AIC=302.04<br>(X^2=285.73, p<.001, X^2/df =4.61, RMSEA=.09, SRMR=.06, CFI=.89, AIC=343.73)	Good fit<br>Good fit
	3F [neg]	X^2=233.31, p<.001, X^2/df =3.76, RMSEA=.08, SRMR=.06, CFI=.93, AIC=317.31<br>(X^2=211.95, p<.001, X^2/df =3.42, RMSEA=.08, SRMR=.05, CFI=.91, AIC=295.95)	Good fit<br>Good fit
	Second order (3[neg]>1 [SCS neg])	X^2=164.09, p<.001, X^2/df = 2.65, RMSEA= 0.06, SRMR= .05, CFI= 0.93, AIC = 291.31<br>(X^2=183.26, p<.001, X^2/df =2.96, RMSEA=.06, SRMR=.05, CFI=.91, AIC=269.95)	Good fit<br>Good fit
	Third order (6>3[pos]>1 [SCS])	X^2=1237.71, p<.001, X^2/df =4.27, RMSEA=.09, SRMR=.12, CFI=.80, AIC=1359.71<br>(X^2=1261.67, p<.001, X^2/df =4.35, RMSEA=.09, SRMR=.12, CFI=.76, AIC=1383.66)	Poor fit<br>Poor fit
	Bifactor (6>1)	X^2=1171.22, p<.001, X^2/df =4.29, RMSEA=.09, SRMR=.13, CFI=.81, AIC=1327.22<br>(X^2=1056.42, p<.001, X^2/df =3.87, RMSEA=.08, SRMR=.11, CFI=.81, AIC=1212.42)	Poor fit<br>Poor fit

Note. CFA = confirmatory factor analysis; SEM = structural equation modelling; ESEM = exploratory structural equation modelling; EFA = exploratory factor analysis; df = degrees of freedom; CFI = Comparative Fit Index; PCFI= parsimony adjusted CFI; TLI = Tucker-Lewis Index; RMSEA = root mean square error of approximation; SRMR = standardized root mean square residual; WRMR = weighted root mean residual; NNFI = non-normed fit index; AIC = Akaike's Information Criterion; ωH = omega hierarchical; ωHS = omega hierarchical subscale; PRV = proportion of reliable variance; ECV = explained common variance. SK = self-kindness, CH = common humanity, MF = mindfulness, SJ = self-judgment, IL = isolation, OI = over- identification;
1F = One-factor model encompassing all 26 SCS items;
2F = Two-factor model encompassing all positive and all negative items, respectively;
3F = Three-factor model reflecting three dimensions of SCS, SK versus SJ, SH versus IL, and MF versus OI;
3F [pos] = Three-factor model reflecting the positive SCS subscales (SK, CH, MF)
3F [neg] = Three –factor model reflecting the negative SCS subscales (SJ, IL, OI)

6F = Six-factor model corresponding to six SCS subscales;
Second order (6>1) = Hierarchical order model with six first order factors and one higher/second order factor;
Second order (6>2) = Hierarchical order model with six first order factors and two second/higher order factors;
Second order (6>3[pos]) = Hierarchical order model with six first order factors and three second/higher order factors, SK, CH, MF
Second order (3[pos]>1 [SCS pos]) = Hierarchical order model with three first order positive SCS factors (SK, CH, MF) and one second/higher order factor (self-compassion)
Second order (3[neg]>1 [SCS neg]) = Hierarchical order model with three first order negative SCS factors (SJ, IL, OI) and one second/higher order factor (self-criticism)
Third order (6>3[pos]>1 [SCS]) = Hierarchical order model with six first order factors (six SCS subscales), three second order factors (SK, CH, MF) and one third order factor (SCS)
Bifactor (6>1) = model with six group factors [SCS subscales] and one target/general factor [overall SCS].
$^{+}$Model fit qualifiers phrased by current authors, but reflecting the evaluation of the model fit as reported in the original study; Good and Adequate fit = in agreement with a priori set criteria; Acceptable fit = somewhat below the a priori set criteria, but still judged as acceptable by the authors. The authors of the original studies differed in their choice of cut-offs and interpretation of their findings.
