# Supplementary material for: Structural validation of the Self-Compassion Scale with a German general population sample
Source: PLoS One. 2018 Feb 6;13(2):e0190771. doi: 10.1371/journal.pone.0190771 (PMC5800544; doi:10.1371/journal.pone.0190771)
Supplement: S2 Table — (PDF) [file pone.0190771.s002.pdf]

S 2: Distribution of the Self-Compassion Scale items to three positive arbitrary and three negative arbitrary factors

We separated the SCS items into positively phrased and negatively phrased. We assigned the positively phrased items to three arbitrarily chosen factors and the negatively phrased SCS items to another three also arbitrarily chosen factors. We limited the number of items per factor to match the original number of items per positive versus negative subscales.

The positively phrased items which correspond to the SCS subscales of self-kindness (2, 19, 23, 26), common humanity (3, 7, 10, 15), and mindfulness (9, 14, 17, 22), were assigned to three arbitrary factors as follows: the first self-kindness item (item 5) was assigned to the arbitrary factor 1; the second self-kindness item (item 12) was assigned to the arbitrary factor 2; the third self-kindness item (item 19) was assigned to the arbitrary factor 3; the fourth self-kindness item (item 23) to arbitrary factor 1; the fifth self-kindness item (item 26) to the arbitrary factor 2. At this point, all of the five self-kindness items had been re-assigned and we started re-assigning the items of the common humanity scale. The first common humanity item (item 3) was assigned to the next factor in line, i.e., arbitrary factor 3, the second common humanity item (item 7) was assigned to arbitrary factor 1, the third common humanity item (item 10) to arbitrary factor 2 and so forth until the items of all three positive SCS subscales had been assigned consecutively and one at a time to the 3 arbitrary positive factors.

- Factor 1: items 5, 12, 19, 23, 26
- Factor 2: items 3, 7, 10, 15
- Factor 3: 9, 14, 17, 22

The same procedure was followed for all of the negatively phrased SCS items, i.e., the items of the SCS subscales self-judgment (1, 8, 11, 16, 21), isolation (4, 13, 18, 25), and over-identification (2, 6, 20, 24).

- Factor 4: items 1, 8, 11, 16, 21
- Factor 5: items 4, 13, 18, 25
- Factor 6: items: 2, 6, 20, 24

This procedure resulted in two factors (one positive and one negative) with 5 items each, and four factors (two positive and two negative) with four items each, whereby no more than two items of an original SCS subscale was assigned to any arbitrary factor. The item distribution for each of the new six factors is presented in the table below.

New Factors (n of items)	Item numbers (and original SCS subscale assignment)
Positive factors	
Factor 1 (5)	5 (SK), 23 (SK), 7 (CH), 9 (MF), 22 (MF)
Factor 2 (4)	12 (SK), 26 (SK), 10 (CH), 14 (MF)
Factor 3 (4)	19 (SK), 3 (CH), 15 (CH), 17 (M)
Negative factors	
Factor 4 (5)	1 (SJ), 16 (SJ), 13 (IL), 2 (OI), 24 (OI)
Factor 5 (4)	8 (SJ), 21 (SJ), 18 (IL), 6 (OI)
Factor 6 (4)	11 (SJ), 4 (IL), 25 (IL), 20 (OI)

Note. SK-self-kindness, CH=common humanity, MF=mindfulness, SJ=self-judgment, IL=isolation, OI= over-identification
