# Supplementary material for: Structural validation of the Self-Compassion Scale with a German general population sample
Source: PLoS One. 2018 Feb 6;13(2):e0190771. doi: 10.1371/journal.pone.0190771 (PMC5800544; doi:10.1371/journal.pone.0190771)
Supplement: S3 Table — (PDF) [file pone.0190771.s003.pdf]

S 3: Factor Loadings and Confidence Intervals (CI) for the Six-factor Structure of the Self-Compassion Scale

Item	Factor loading	95% CI
**SCS Self Kindness Factor**		
SCS 5	0.66	[0.64, 0.69]
SCS 12	0.77	[0.75, 0.79]
SCS 19	0.66	[0.63, 0.68]
SCS 23	0.57	[0.54, 0.59]
SCS 26	0.76	[0.74, 0.78]
**SCS Self Judgment Factor**		
SCS 1	0.59	[0.56, 0.62]
SCS 8	0.56	[0.53, 0.59]
SCS 11	0.65	[0.62, 0.67]
SCS 16	0.77	[0.75, 0.79]
SCS 21	0.63	[0.60, 0.66]
**SCS Common Humanity Factor**		
SCS 3	0.66	[0.64, 0.69]
SCS 7	0.63	[0.60, 0.65]
SCS 10	0.66	[0.63, 0.69]
SCS 15	0.66	[0.63, 0.69]
**SCS Isolation Factor**		
SCS 4	0.76	[0.73, 0.78]
SCS 13	0.65	[0.62, 0.67]
SCS 18	0.64	[0.61, 0.67]
SCS 25	0.76	[0.74, 0.78]

**SCS Mindfulness Factor**		
SCS 9	0.74	[0.71, 0.76]
SCS 14	0.75	[0.73, 0.77]
SCS 17	0.71	[0.69, 0.73]
SCS 22	0.67	[0.65, 0.70]
**SCS Over-identification Factor**		
SCS 2	0.71	[0.68, 0.73]
SCS 6	0.72	[0.70, 0.75]
SCS 20	0.60	[0.57, 0.62]
SCS 24	0.59	[0.57, 0.62]
